# *ROSEA1*-Based Visual Selection Reduces Plant Regeneration and Alters Developmental Regulator Expression

**DOI:** 10.3390/plants15132004

**Published:** 2026-06-28

**Authors:** Tao Jiang, Sameena Ejaz Tanwir, Fangchen Liu, Wisnu Handoyo Ardi, Yeyen Novitasari, Sandy Zammar, Fida Hussain, Heqiang Huo

**Affiliations:** 1 Crop Transformation Center, Department of Horticulture Sciences, IFAS, University of Florida, Gainesville, FL 32611, USA; taojiang@ufl.edu (T.J.); tanwir.s@ufl.edu (S.E.T.); liufangchen@ufl.edu (F.L.); w.handoyoardi@ufl.edu (W.H.A.); 2 Mid-Florida Research & Education Center, IFAS, University of Florida, Apopka, FL 32703, USA; yeyen.novitasari@ufl.edu (Y.N.); szammar2@outlook.com (S.Z.); fida1385@gmail.com (F.H.); 3 Wheat Research Institute, Ayub Agricultural Research Institute, Faisalabad University of Agriculture, Faisalabad 38000, Pakistan

**Keywords:** *ROSEA1*, anthocyanin biosynthesis, regeneration capacity, metabolic flux

## Abstract

Anthocyanin-based visual reporters enable rapid, non-destructive identification of transgenic tissues, but their pigment output may not be physiologically neutral during organogenesis, raising concerns about their suitability as selection markers in transformation pipelines. To address this, we examined the regeneration performance of constitutive *ROSEA1* expression across four eudicot species from three families: tomato, petunia, begonia, and marigold. Stable *ROSEA1*-overexpressing lines were evaluated in tomato and petunia, whereas transformation-stage assays were performed in begonia and marigold. Regeneration frequencies were quantified relative to controls, and transcript-level changes were assessed for anthocyanin biosynthetic genes and regeneration regulators; complementation assays with *PLT5* co-expression and hormonal manipulations were also performed. In tomato and petunia, *ROSEA1*-overexpressing lines exhibited reduced regeneration frequencies and elevated anthocyanin accumulation, with two independent petunia lines showing that stronger pigment activation corresponded to more severe regeneration defects. In tomato, *ROSEA1* coordinately upregulated *CHI*, *F3H*, *F3′5′H*, and *DFR* and was associated with reduced expression of *PLT5*, *WUS*, and *LBD16* during early regeneration. *PLT5* co-expression partially restored regeneration with modestly reduced pigmentation, indicating that pigment output alone does not fully explain the phenotype. The penalty extended to begonia and marigold, with genotype- and hormone-dependent severity in marigold. These findings indicate a context-dependent regeneration penalty associated with constitutive *ROSEA1* expression and suggest that hormone optimization, genotype selection, and developmental support may help mitigate this limitation in some contexts.

## 1. Introduction

Visual reporters that produce pigmentation in plant cells provide a convenient means to identify transgenic events without destructive assays or specialized equipment. Among these, the R2R3-MYB transcription factor *ROSEA1* from *Antirrhinum majus* has become a widely adopted marker owing to its ability to induce robust anthocyanin accumulation across diverse plant species [1,2]. Unlike conventional reporters such as β-glucuronidase (GUS), which require destructive histochemical assays and overnight incubation [3], or green fluorescent protein (GFP), which necessitates fluorescence microscopy and UV illumination [4,5], *ROSEA1* and related anthocyanin-inducing transcription factors enable the real-time, non-destructive visual tracking of transgenic tissues. This class of visual marker has been incorporated into transformation vectors for applications ranging from tracking viral infections to visual selection in rubber tree and hairy root systems [2,6,7]. Despite this widespread adoption, the effects of *ROSEA1* expression on plant regeneration capacity, a critical determinant of transformation success, remain poorly characterized.

*ROSEA1* activates anthocyanin biosynthesis by forming a regulatory complex with endogenous basic helix-loop-helix (bHLH) and WD40 proteins, collectively known as the MYB-bHLH-WD40 (MBW) complex [8,9]. This highly conserved regulatory module coordinates the expression of structural genes throughout the flavonoid pathway, including chalcone synthase (*CHS*), chalcone isomerase (*CHI*), flavanone 3-hydroxylase (*F3H*), dihydroflavonol 4-reductase (*DFR*), and anthocyanidin synthase (*ANS*) [10,11]. The broad conservation of endogenous bHLH partners across angiosperms enables *ROSEA1* expression alone to induce visible pigmentation in most plant species [12,13]. Landmark work demonstrated that co-expression of *ROSEA1* and its bHLH partner, *DELILA*, produced intensely purple tomato fruits with anthocyanin levels comparable to those of blueberries, thereby establishing the metabolic engineering potential of these transcription factors [1]. However, strong constitutive activation of flavonoid biosynthesis may also impose developmental and metabolic consequences beyond pigmentation itself.

Anthocyanin production requires substantial investment of carbon skeletons derived from phenylalanine and malonyl-CoA, potentially diverting metabolic flux from primary pathways and other phenylpropanoid branches, including lignin biosynthesis and protein synthesis [14,15,16]. Its biosynthesis and vacuolar sequestration also require multiple enzymatic and tailoring steps, representing a considerable cellular resource commitment [14,17]. Trade-offs among anthocyanin accumulation, vegetative growth, reproductive output, and stress tolerance have been documented across diverse species [15,18]. These observations suggest that strong anthocyanin-promoting programs may interfere with the regenerative capacity of cultured explants, in which cellular resources must support both pigment biosynthesis and shoot organogenesis.

The potential impact of anthocyanin accumulation on regeneration is particularly significant, as regeneration capacity remains a major bottleneck in plant transformation. Genetic transformation underpins modern plant biotechnology, enabling functional genomics, precision breeding, and CRISPR/Cas-based genome editing [19,20]. The success of any transformation pipeline depends on two sequential processes: delivery of foreign DNA into plant cells and subsequent regeneration of whole plants from transformed tissues [19,21]. While DNA delivery methods have been substantially optimized, regeneration recalcitrance, the inability of plant tissues to form organized structures under in vitro conditions or after genetic transformation, continues to restrict the scope of genetic engineering across legumes, cereals, woody species, and many elite cultivars [22,23,24]. Moreover, the genotype-specificity of regeneration responses means that protocols optimized for one cultivar frequently fail when applied to related genotypes, and genome-editing tools can be applied only to the narrow subset of genotypes amenable to regeneration [25,26].

De novo shoot organogenesis proceeds through a coordinated developmental sequence governed by hormonal and transcriptional programs. The classical work of Skoog and Miller established a foundational framework in which the relative balance of exogenous auxin and cytokinin in culture media is associated with different morphogenic outcomes, with cytokinin-rich conditions generally favoring shoot formation and auxin-rich conditions favoring root development [27]. This framework has strongly influenced the development of callus-induction media (CIM) and shoot-induction media (SIM) used in plant tissue culture. However, regeneration outcomes are not determined by exogenous hormone balance alone and depend on endogenous hormonal status, tissue identity, developmental competence, and species- or genotype-specific regulatory contexts [28,29]. At the molecular level, key transcription factors orchestrate regeneration, such as *WUSCHEL* (*WUS*), *PLETHORA* (*PLT3*, *PLT5*, *PLT7*), and *LATERAL ORGAN BOUNDARIES DOMAIN* factors (*LBD16*, *LBD29*), whose perturbation is associated with impaired regeneration outcomes [30,31,32,33,34,35]. Notably, several lines of evidence suggest potential mechanistic links between anthocyanin biosynthesis and regeneration pathways: cytokinin has been shown to stimulate both shoot organogenesis and anthocyanin accumulation [36,37], while the phenylpropanoid pathway shares metabolic precursors with auxin biosynthesis [38]. Consequently, constitutive *ROSEA1* expression could influence regeneration through metabolic competition, hormonal crosstalk, or transcriptional interference with key developmental networks.

Despite the growing deployment of *ROSEA1* and related anthocyanin-inducing MYB factors as visible reporters in transformation systems, their effects on regeneration have not been systematically evaluated [2,39,40]. Existing evidence suggests context-dependent outcomes. In cassava, anthocyanin induction via the R2R3-MYB *HbAN1* did not significantly affect regeneration [41]. In several dicots, anthocyanin-based markers such as MYB10 have been reported to be compatible with organogenesis during transformation, including in apple, strawberry, and potato [39]. By comparison, maize anthocyanin regulators were first established as visible transformation markers in maize with the *Lc* gene [42], and maize *C1/B-peru* regulators were later shown to induce pigmentation in transformed wheat tissues [43], while related anthocyanin-based visible-marker applications have also been reported in sugarcane [44]. These studies show that anthocyanin-inducing regulators have been applied as visible markers across several systems, although their developmental consequences for regeneration were not systematically evaluated. Accordingly, no study has yet quantified the magnitude of a regeneration penalty across multiple species, profiled underlying transcriptional changes, or tested whether such effects extend across phylogenetically diverse lineages. This gap creates uncertainty when selecting visual markers, particularly in regeneration-sensitive genotypes. Defining the magnitude and basis of any such penalty would help guide mitigation strategies, such as hormone optimization, genotype selection, or developmental support, while preserving the practical advantages of visual selection.

Here, we show that constitutive *ROSEA1* expression is associated with reduced regeneration across four plant species examined in stable transgenic lines and transformation-stage assays. These findings indicate that *ROSEA1*-based visual selection can impose a context-dependent regeneration penalty. We further highlight hormone optimization, genotype selection, and developmental support as potential strategies that may help mitigate this trade-off in some contexts.

## 2. Results

### 2.1. ROSEA1 Expression Compromises Regeneration During Tomato Transformation and in Stable Transgenic Lines

**Figure 1:**
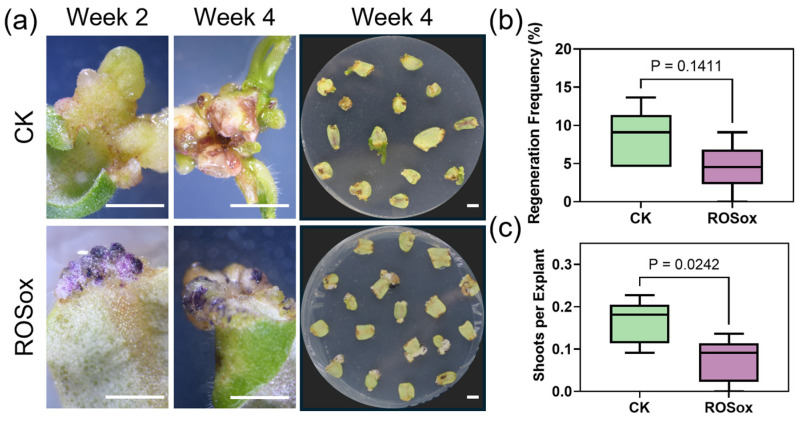
*ROSEA1* reduces plant regeneration during transformation in tomato. (**a**) Representative phenotypes of tomato explants during *Agrobacterium*-mediated transformation under control (CK) and *ROSEA1* (ROSox) treatments. CK explants regenerated green shoots without visible anthocyanin accumulation, whereas ROSox explants developed purple anthocyanin pigmentation and exhibited reduced regeneration. Left and middle panels show close-up views at week 2 and week 4, respectively; right panels show representative Petri-dish overviews at week 4. (**b**,**c**) Regeneration frequency (%) (**b**) and shoots per explant (**c**) in CK (green) and ROSox (purple) treatments. Data are presented as mean ± s.d. (*n* = 4 biological replicates; 20 explants per replicate). Statistical significance was assessed using two-tailed Student’s *t*-tests; *p*-values are indicated. Scale bars, 5 mm (close-ups in (**a**)) and 1 cm (Petri dishes in (**a**)).

To determine whether *ROSEA1*-based visual selection influences regeneration during the transformation process itself, tomato explants were subjected to *Agrobacterium*-mediated transformation with a *ROSEA1*-containing construct and compared with empty vector controls transformed in parallel under identical culture conditions. The overexpressing *ROSEA1* (ROSox) transformation tissues developed purple pigmentation in regenerating regions, visible as discrete purple foci emerging from the wound sites, whereas control (CK) explants remained green throughout the culture period (Figure 1a). ROSox explants produced fewer visible regenerative structures than CK explants, as evidenced by both magnified views and plate-level observations. The proportion of regenerating explants was numerically 44.4% lower in ROSox than in CK (Figure 1b), whereas shoots per explant were significantly reduced by 55.5% in ROSox (Figure 1c).

**Figure 2:**
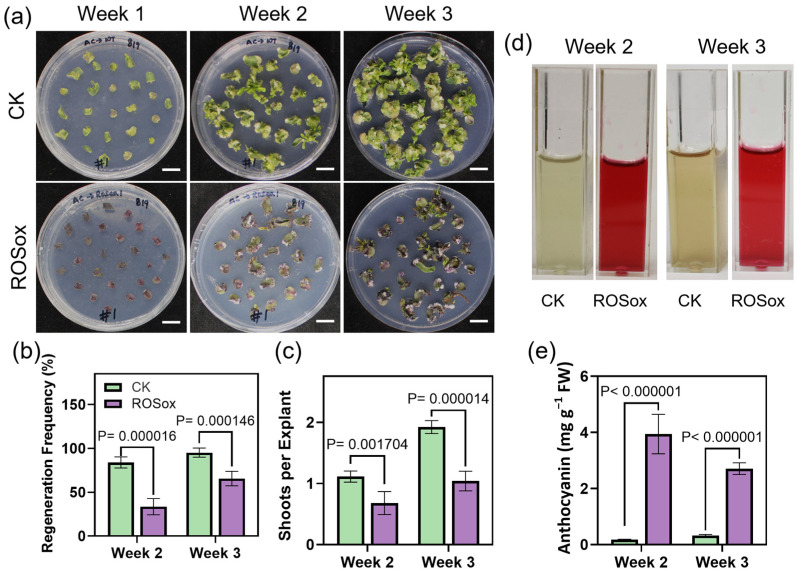
*ROSEA1* overexpression reduces regeneration in stable transgenic tomato lines. (**a**) Representative regeneration phenotypes of stable transgenic tomato lines overexpressing *ROSEA1* (ROSox; anthocyanin-pigmented tissues) and control lines (CK) from week 1 to week 3 after regeneration induction. Scale bars, 1 cm. (**b**,**c**) Regeneration frequency (%) (**b**) and shoots per explant (**c**) in CK (green) and ROSox (purple) lines at week 2 and week 3. Data are presented as mean ± s.d. (*n* = 4 biological replicates; 20 explants per replicate). (**d**) Representative anthocyanin extracts from CK and ROSox tissues collected at week 2 and week 3. (**e**) Anthocyanin content (mg g^−1^ FW) in CK and ROSox tissues at week 2 and week 3 (*n* = 4 biological replicates; three technical replicates per biological replicate). Statistical significance was assessed using two-tailed Student’s *t*-tests; *p*-values are indicated.

To determine whether the regeneration deficit persisted beyond the transformation stage and reflected a stable phenotype associated with constitutive *ROSEA1* expression, stable transgenic tomato lines ROSox were generated and subjected to regeneration assays. ROSox lines exhibited sustained purple pigmentation throughout three weeks of culture on regeneration medium, with coloration intensifying over time (Figure 2a). Shoot emergence in ROSox lagged behind CK from the earliest time point examined, and this difference widened from week 1 to week 3 (Figure 2a). Quantification of regeneration parameters revealed substantial differences between genotypes. Regeneration frequency at week 2 was reduced by 60.0% in ROSox relative to CK, representing the maximum divergence (Figure 2b). By week 3, regeneration frequency in ROSox had partially recovered but remained 43.5% lower than CK (Figure 2b). Shoot production per explant was significantly reduced, declining by 52.4% at week 2 and by 42.6% at week 3 in ROSox relative to CK (Figure 2c). To confirm that the pigmentation phenotype reflected elevated anthocyanin biosynthesis, pigments were extracted from regenerated tissues and quantified. Anthocyanin extracts from ROSox were intensely colored compared with the pale solutions obtained from CK (Figure 2d). Spectrophotometric quantification confirmed that anthocyanin levels were 20.7-fold and 7.6-fold higher in ROSox than CK at weeks 2 and 3, respectively (Figure 2e).

To test whether the regeneration penalty was specific to *ROSEA1* or a general consequence of anthocyanin pathway activation, stable transgenic tomato lines overexpressing the bHLH transcription factor *DELILA* (DELox) were evaluated in parallel (Figure S1). No obvious reduction in shoot production was detected in DELox lines under the conditions tested (Figure S1a,c). At week 3, DELox tissues accumulated 1.74-fold more anthocyanin than CK (Figure S1b), confirming that *DELILA* also enhanced anthocyanin biosynthesis, albeit to a substantially lower level than *ROSEA1*. In contrast to ROSox, however, DELox did not exhibit reduced regenerative capacity; shoot production was 13.3% higher than in CK at the same stage, although this difference was not statistically significant (Figure S1c). These results indicate suggest that anthocyanin accumulation alone does not fully explain the regeneration phenotype and that regulator identity and/or the magnitude of pathway activation may also contribute to the difference observed between DELox and ROSox.

### 2.2. ROSEA1 Is Associated with Altered Gene Expression During Regeneration, Including Anthocyanin-Pathway Induction and Changes in Regeneration-Associated Regulators

**Figure 3:**
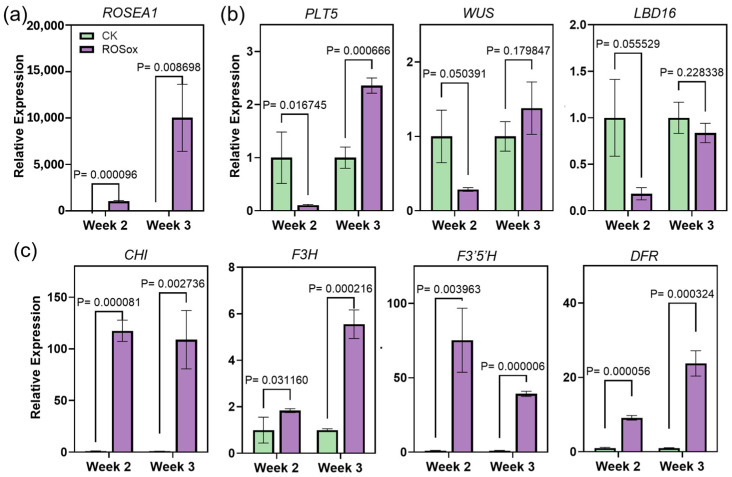
*ROSEA1* is associated with increased expression of anthocyanin-biosynthesis genes and altered expression of regeneration-associated genes in tomato. (**a**) qRT–PCR analysis of *ROSEA1* expression level in control (CK; green) and *ROSEA1* overexpression (ROSox; purple) tomato lines at week 2 and week 3. (**b**) qRT–PCR analysis of regeneration-associated genes, including *PLT5*, *WUS* and *LBD16* in CK and ROSox lines at week 2 and week 3. (**c**) qRT–PCR analysis of anthocyanin-biosynthesis genes, including *CHI*, *F3H*, *F3′5′H* and *DFR* in CK and ROSox lines at week 2 and week 3. Expression values were normalized to the reference gene *ACTIN2*. Data are presented as mean ± s.d. (*n* = 3 biological replicates; three technical replicates per biological replicate). Statistical significance was assessed using two-tailed Student’s *t*-tests; *p*-values are indicated.

Quantitative real-time PCR (qRT-PCR) was performed on ROSox and CK tissues harvested at weeks 2 and 3 to characterize the transcriptional changes underlying the regeneration deficit. These time points were selected because week 2 corresponded to the period of maximum divergence in regeneration frequency between genotypes, whereas week 3 captured the partial recovery phase. *ROSEA1* transcript abundance in ROSox was readily detected at both time points and remained substantially higher than in CK (Figure 3a), indicating sustained transgene expression throughout the culture period.

Anthocyanin pathway genes were coordinately activated in ROSox tissues. *CHI* increased 117.5-fold at week 2 and 108.9-fold at week 3 relative to CK. *DFR* increased to 9.1-fold at week 2 and 23.8-fold at week 3 (Figure 3c). *F3H* exhibited a more gradual response, increasing 1.8-fold at week 2 and 5.6-fold at week 3. *F3′5′H* (*HF1*) increased 75.3-fold at week 2 and 39.2-fold at week 3 (Figure 3c). Together, these data show coordinated induction of multiple anthocyanin-pathway genes in ROSox tissues.

Given the reduced regeneration capacity observed in ROSox lines, the expression of genes encoding key regulators of shoot regeneration was examined. *PLT5*, *WUS*, and *LBD16* were therefore analyzed in parallel with the anthocyanin-pathway genes. In contrast to the anthocyanin-related genes, regeneration-associated regulators showed their lowest expression levels at the early time point. At week 2, *PLT5* expression was significantly reduced by 89.3%, whereas *WUS* and *LBD16* showed numerically lower expression than CK (Figure 3b). By week 3, *PLT5* rebounded to 2.4-fold above CK levels, while *WUS* trended upward and *LBD16* remained slightly lower than CK, although these latter differences were not statistically significant (Figure 3b). This expression pattern coincided with the stage at which the regeneration difference between genotypes was greatest (Figure 2b). Overall, ROSox tissues showed sustained anthocyanin-pathway induction together with a shift in the expression pattern of regeneration-associated regulators across the two sampled stages.

**Figure 4:**
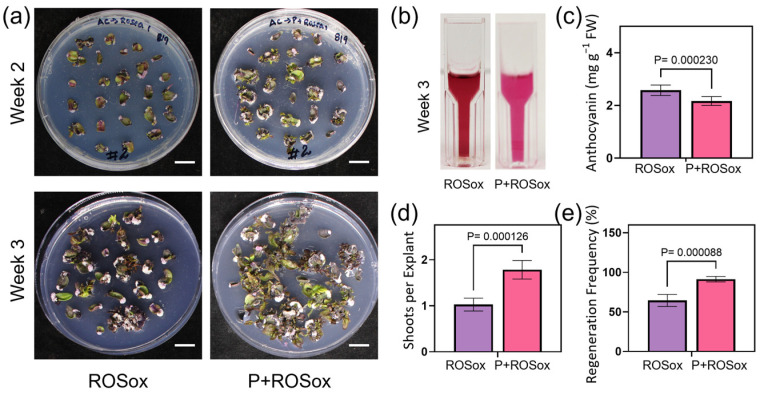
Co-expression of *PLT5* is associated with partial recovery of regeneration-related traits in stable transgenic tomato lines overexpressing *ROSEA1*. (**a**) Representative regeneration phenotypes of stable transgenic tomato lines overexpressing *ROSEA1* alone (ROSox; anthocyanin-pigmented tissues) or co-expressing *PLT5* and *ROSEA1* (P + ROSox) at week 2 and week 3 after regeneration induction. Scale bars, 1 cm. (**b**) Representative anthocyanin extracts from ROSox and P + ROSox tissues collected at week 3. (**c**) Anthocyanin content (mg g^−1^ FW) in ROSox and P + ROSox tissues. (**d**,**e**) Shoots per explant (**d**) and regeneration frequency (%) (**e**) in ROSox and P + ROSox lines. Data are presented as mean ± s.d. (*n* = 4 biological replicates; 20 explants per replicate). Statistical significance was assessed using two-tailed Student’s *t*-tests; *p*-values are indicated.

To test whether reinforcement of the regeneration program was associated with partial recovery of the ROSox phenotype, stable tomato lines co-expressing *PLT5* and *ROSEA1* (P + ROSox) were evaluated alongside ROSox lines (Figure 4). Relative to ROSox, P + ROSox lines showed a 73.7% increase in shoots per explant and a 41.9% increase in regeneration frequency, indicating partial recovery of regeneration-associated traits (Figure 4d,e). In contrast, anthocyanin accumulation in P + ROSox tissues was reduced by 18.8% relative to ROSox (Figure 4b,c), showing that the improved regeneration phenotype was accompanied by only a modest reduction in pigment output. These results indicate that pigment output alone does not fully explain the phenotype and that *PLT5* co-expression was associated with partial phenotypic rescue, without establishing *PLT5* as the sole mediator of the regeneration response.

### 2.3. The Regeneration Penalty Associated with ROSEA1 Is Observed Across Diverse Plant Species

**Figure 5:**
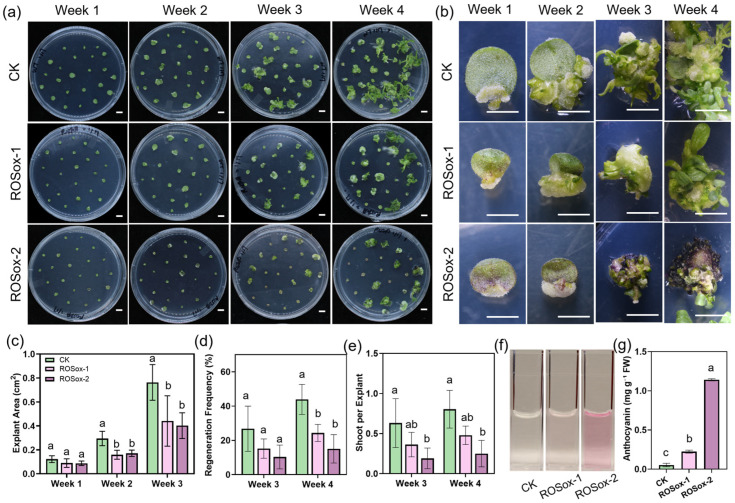
*ROSEA1* overexpression is associated with reduced regenerative output in stable transgenic petunia lines. (**a**) Time-course of regeneration from week 1 to week 4 in petunia explants for control (CK, **top**) and two independent *ROSEA1* overexpression lines (ROSox-1, **middle**; ROSox-2, **bottom**), showing reduced plantlet formation in ROSox. (**b**) Representative close-up views of regenerating tissues from week 1 to week 4 for CK, ROSox-1, and ROSox-2. (**c**) Explant area quantified from week 1 to week 3 (CK, green; ROSox-1, light purple; ROSox-2, dark purple) (*n* = 20 explants measured per genotype at each time point). (**d**) Regeneration frequency (%) at week 3 and week 4 in CK, ROSox-1 and ROSox-2. (**e**) Regenerated shoots per explant at week 3 and week 4 in CK, ROSox-1, and ROSox-2 ((**d**,**e**): *n* = 6 biological replicates; 20 explants per replicate). (**f**) Representative anthocyanin extracts from CK, ROSox-1, and ROSox-2 tissues collected at week 4. (**g**) Anthocyanin content (mg g^−1^ FW) at week 4 in CK, ROSox-1, and ROSox-2 tissues (*n* = 4 biological replicates; 3 technical replicates per biological replicate). Data are presented as mean ± s.d. In panels (**c**–**e**,**g**), different lowercase letters above the bars indicate significant differences among groups at each time point according to Tukey’s multiple-comparisons test (*p* < 0.05). Scale bars, 1 cm (**a**) and 5 mm (**b**).

To test whether the regeneration deficit observed in tomato reflects a broader consequence of *ROSEA1* expression, we examined two independent stable petunia ROSox lines with distinct anthocyanin intensities (ROSox-1, moderate; ROSox-2, strong) in time-course assays (Figure 5 and Figure S2). In an initial screening experiment, ROSox-2 accumulated visible purple pigmentation throughout culture, whereas control (CK) explants remained green (Figure S2a,b). ROSox-2 cultures consistently produced fewer regenerants and exhibited delayed shoot progression relative to CK over a 1-to-3-week culture window (Figure S2a,b). Quantification of explant expansion supported this phenotype: explant area in ROSox-2 lagged behind CK at all time points, being reduced by 18.3% at week 1, 32.2% at week 2, and 41.6% at week 3 relative to CK (Figure S2c). Regeneration frequency was reduced by 47.2% at week 2 and remained 40.0% lower at week 3 (Figure S2d). Anthocyanin extracts from week-3 tissues were visibly pink in ROSox-2 but colorless in CK, with pigment levels 21.0-fold higher than CK (Figure S2e,f).

We then directly compared ROSox-1 and ROSox-2 in a time-course assay extending to week 4 (Figure 5a,b). Both lines maintained purple pigmentation throughout culture and produced fewer regenerants with delayed shoot progression at the plate level relative to CK (Figure 5a,b). Quantification of explant expansion confirmed that reduced tissue growth emerged early and persisted: compared with CK, mean explant area was reduced by 27.6% in ROSox-1 and 29.2% in ROSox-2 at week 1, by 46.2% in ROSox-1 and 42.1% in ROSox-2 at week 2, and by 42.8% in ROSox-1 and 47.1% in ROSox-2 at week 3 (Figure 5c). Regeneration frequency was also lower in both ROSox lines than in CK at weeks 3 and 4 (Figure 5d). By week 4, ROSox-1 remained 29.3% lower than CK, whereas ROSox-2 remained 64.1% lower. Likewise, regenerated shoots per explant at week 4 were reduced most strongly in ROSox-2, with a milder effect in ROSox-1 (Figure 5e). Biochemical analysis confirmed the intended pigment gradient: anthocyanin content at week 4 was 4.3-fold higher in ROSox-1 and 21.7-fold higher in ROSox-2 relative to CK (Figure 5f,g). These results support a *ROSEA1*-associated reduction in regenerative output in petunia and indicate that the more strongly pigmented independent line showed the more severe phenotype under the conditions tested.

**Figure 6:**
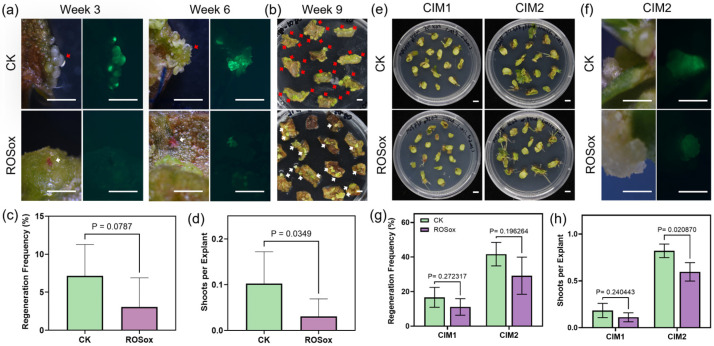
*ROSEA1* is associated with reduced regenerative output during transformation in begonia and marigold. (**a**) Representative close-up bright-field and fluorescence images of transforming begonia explants under control (CK, **top**) and *ROSEA1* (ROSox, **bottom**) treatments at weeks 3 and 6. (**b**) Representative Petri-dish overviews at week 9 for begonia CK (**top**) and ROSox (**bottom**), showing reduced regeneration in ROSox; red and white arrows indicate regenerating explants in CK and ROSox, respectively. (**c**,**d**) Regeneration frequency (%) (**c**) and shoots per explant (**d**) in begonia CK (green) and ROSox (purple) treatments. Data are presented as mean ± s.d. (*n* = 4 biological replicates; 15 explants per replicate). (**e**) Representative Petri dishes of marigold ‘Marvel II Yellow’ explants during *Agrobacterium*-mediated transformation under CK and ROSox treatments on two callus-induction media: CIM1 (0.5 mg L^−1^ NAA + 0.75 mg L^−1^ BAP) and CIM2 (0.5 mg L^−1^ NAA + 2.0 mg L^−1^ BAP), showing reduced regeneration in ROSox. (**f**) Representative bright-field and fluorescence images of regenerating marigold tissues from CK (**top**) and ROSox (**bottom**) explants, showing reporter fluorescence in the corresponding panels. (**g**,**h**), Regeneration frequency (%) (**g**) and shoots per explant (**h**) for marigold CK (green) and ROSox (purple) across CIM1 and CIM2 conditions. Data are presented as mean ± s.d. (*n* = 5 biological replicates; 15 explants per replicate). Statistical significance was assessed using two-tailed Student’s *t*-tests; *p*-values are indicated. Scale bars, 5 mm (**a**,**f**) and 1 cm (**b**,**e**).

Begonia, phylogenetically distant from *Solanaceae* and representing the order Cucurbitales, was included to broaden the taxonomic scope of the analysis and test whether the *ROSEA1*-associated penalty extends beyond the Solanaceae family. *Agrobacterium*-mediated transformation introduced *ROSEA1* into begonia leaf explants, and fluorescence signals together with developing structures were observed in ROSox explants at weeks 3 and 6 (Figure 6a), supporting successful transformation of ROSox explants. At week 9, when regeneration scoring was performed, CK cultures had produced visibly more regenerants than ROSox cultures at the plate level (Figure 6b). Regeneration frequency was numerically 57.4% lower in ROSox than in CK (Figure 6c), while shoots per explant were significantly reduced by 70.1% (Figure 6d). These data indicate reduced regenerative output in begonia ROSox explants under the conditions tested.

To further evaluate context dependence, marigold (*Tagetes erecta*), a member of the *Asteraceae* family, was examined under two callus-induction media differing in cytokinin content. *Agrobacterium*-mediated transformation of marigold ‘Marvel II Yellow’ was carried out under CIM1 (0.5 mg L^−1^ NAA + 0.75 mg L^−1^ BAP) and CIM2 (0.5 mg L^−1^ NAA + 2.0 mg L^−1^ BAP). ROSox explants exhibited reduced regenerative progression relative to CK on both formulations at the plate level (Figure 6e). Reporter fluorescence was detected in regenerating tissues (Figure 6f), supporting successful transformation of both treatments. On CIM1, regeneration frequency was numerically 33.1% lower in ROSox relative to CK, whereas on CIM2, regeneration frequency was 30.9% lower (Figure 6g). Shoot output followed a similar trend, decreasing by 35.2% on CIM1 and by 16.2% on CIM2, although the reduction reached statistical significance only on CIM2 (Figure 6h). These data support a context-dependent *ROSEA1*-associated reduction in regenerative output in marigold, with the apparent effect less pronounced under higher cytokinin conditions.

The attenuation of the regeneration penalty at elevated cytokinin levels prompted us to ask whether further increases could fully offset the *ROSEA1* effect. To test this, ‘Marvel II Yellow’ explants were cultured on CIM3 (0.5 mg L^−1^ NAA + 2.5 mg L^−1^ BAP), which supported substantially higher baseline regeneration than CIM1 or CIM2. Under CIM3 conditions, CK and ROSox cultures appeared macroscopically similar throughout the culture period (Figure S3a). No significant differences were detected between treatments for either regeneration frequency (Figure S3b) or shoots per explant (Figure S3c). These results indicate that the *ROSEA1*-associated phenotype was not detectable under CIM3 conditions.

The *ROSEA1*-associated penalty also appeared to vary with genotype. In ‘Bonanza Yellow’ cultured on CIM1, baseline regeneration was low for both CK and ROSox treatments, and plate phenotypes appeared similar (Figure S3d). Neither regeneration frequency nor shoot output differed significantly between treatments (Figure S3e,f); ROSox values for regeneration showed a slight, non-significant increase relative to CK (Figure S3e,f). The inherently low regeneration capacity of this genotype may have masked additional ROSEA1-associated effects. Taken together, the marigold data supports context dependence across hormone regimes and genotypes under the conditions tested. In these transformation-stage assays, the quantified outcome was reduced regenerative output in *ROSEA1*-expressing explants; the present data do not distinguish among reduced explant survival, altered transformation-stage progression, and reduced organogenic competence.

## 3. Discussion

Visual reporters that enable the non-destructive identification of transgenic events are valuable tools in plant transformation, but their developmental neutrality has rarely been examined systematically. In this study, constitutive *ROSEA1* expression was associated with reduced regeneration across multiple eudicot species, with the magnitude varying by species, genotype, and hormone regime (Figure 1, Figure 2, Figure 5 and Figure 6). In tomato, this phenotype coincided with activation of anthocyanin biosynthetic genes and reduced expression of key regeneration regulators during an early regeneration window (Figure 3), providing mechanistic clues consistent with the observed trade-off.

### 3.1. Possible Bases of the ROSEA1–Regeneration Trade-Off

The data presented here argue against a simple model in which anthocyanin accumulation alone directly suppresses regeneration. This distinction is most clearly illustrated by the *DELILA* comparison: although DELox tomato lines accumulated 1.74-fold as much anthocyanin as the control at week 3, they did not exhibit reduced regenerative capacity (Figure S1). By contrast, ROSox lines showed substantially greater anthocyanin induction, reaching 20.7-fold above the control at week 2 and 7.6-fold at week 3, accompanied by a marked reduction in regeneration (Figure 2). A similar dosage-dependent pattern was observed in petunia, where the more intensely pigmented ROSox-2 line exhibited a stronger regeneration defect than the moderately pigmented ROSox-1 (Figure 5). These comparisons indicate that the severity of the phenotype may correlate more closely with the extent of *ROSEA1*-driven pathway activation than with pigment accumulation per se. This interpretation is consistent with the biology of *ROSEA1* in *Antirrhinum*, where it functions as an R2R3-MYB regulator of floral pigmentation intensity and patterning rather than as a canonical developmental regulator [8], and with previous reports that ectopic *ROSEA1* expression can alter cellular programs beyond pigmentation, including abiotic stress responses [12].

A key line of support comes from the transcriptional data. During the competence acquisition phase, *ROSEA1* overexpression was associated with reduced expression of *PLT5*, *WUS*, and *LBD16* (Figure 3b), coinciding with the developmental stage at which the divergence in regeneration between ROSox and the control was maximal (Figure 2 and Figure 3). This temporal overlap is notable because these genes occupy central positions in regeneration: PLT factors promote the acquisition of pluripotency [32], *WUS* is required for shoot formation [30], and *LBD16* contributes to the establishment of organogenic competence upstream of *WUS* [35,45]. Consistent with this framework, lines co-expressing *PLT5* and *ROSEA1* (P + ROSox) exhibited a 73.7% increase in shoots per explant and a 41.9% increase in regeneration frequency relative to ROSox, whereas anthocyanin levels declined by only 18.8% (Figure 4). The disproportionate recovery of regeneration relative to pigment reduction is consistent with the possibility that the *ROSEA1*-associated penalty is not driven solely by anthocyanin accumulation but instead may involve altered regeneration-promoting developmental pathways that can be partially restored by *PLT5*. Because de novo shoot organogenesis proceeds through multiple stage-specific steps, strong constitutive *ROSEA1* activity may be most detrimental during a relatively limited early regeneration window. This interpretation is consistent with current views of regeneration as a dynamic, multistep process shaped by tissue competence and endogenous regulatory status, rather than by exogenous culture conditions alone [29,46]. It is also compatible with previous evidence that broader constitutive expression can impose stronger developmental effects than more spatially restricted promoter activity [40], raising the possibility that prolonged *ROSEA1* activity beyond the stage at which visual selection is required could reduce regenerative capacity. Importantly, these data do not indicate that *ROSEA1* itself directly regulates regeneration; rather, ectopic *ROSEA1* activity may perturb the transcriptional environment required for shoot induction, thereby indirectly suppressing the regeneration program.

One possible explanation underlying this effect is metabolic burden. Anthocyanin biosynthesis requires a substantial investment of phenylalanine-derived carbon skeletons and malonyl-CoA, potentially diverting metabolic flux from pathways that support cell proliferation and differentiation [15]. Anthocyanin accumulation is closely linked to growth, energy allocation, and environmental responsiveness rather than representing a metabolically neutral output [47], and engineered high-anthocyanin tomato genotypes have been reported to show reduced vegetative growth and yield [48]. These studies focused on whole-plant growth rather than regeneration-stage tissues, but they are broadly consistent with our observation that the regeneration penalty became most apparent when anthocyanin induction was especially pronounced (Figure 2, Figure 5 and Figure S1), compatible with a threshold-like effect. However, the partial rescue observed in P + ROSox lines, in which regeneration improved markedly despite only modest reductions in pigment output (Figure 4), suggests that metabolic cost alone may not fully account for the phenotype.

A complementary explanation is broader regulatory pleiotropy. As a transcription factor, *ROSEA1* may influence pathways beyond anthocyanin biosynthesis. Indeed, transcriptomic and metabolomic studies of tomato expressing snapdragon anthocyanin regulators have suggested widespread reprogramming across flavonoid and phenylpropanoid networks rather than a confined increase in pigment accumulation [49]. The coordinate suppression of *PLT5*, *WUS*, and *LBD16* (Figure 3b) may therefore reflect either a shared upstream perturbation or parallel interference across multiple developmental pathways. Supporting this view, callus cultures of *Nicotiana benthamiana* expressing *ROSEA1* and *DELILA* under the stronger constitutive 35S promoter showed enhanced pigmentation but reduced shoot formation compared with those driven by the weaker, petal-specific FBP1 promoter [40]. These observations are consistent with the possibility that stronger transgene activity is associated with a more pronounced regeneration penalty.

Hormone-related processes may also contribute to the observed phenotype. Although there is currently no evidence that *ROSEA1* directly represses *PLT5*, *WUS*, or *LBD16*, the biological plausibility of such a link is considerable because anthocyanin-associated regulation is closely integrated with sugar signaling and multiple phytohormone pathways [37,47,50]. Notably, elevated cytokinin largely alleviated the regeneration penalty in marigold (Figure 6e–h and Figure S3), showing that exogenous hormone supply can partially mitigate the *ROSEA1*-associated phenotype, consistent with the broader principle that regeneration barriers can often be mitigated through hormonal optimization [46,51]. However, the present data do not demonstrate that *ROSEA1* directly alters hormone signaling, and hormone-related effects are therefore best regarded as a testable hypothesis rather than an established explanation.

Taken together, the present data are consistent with a model in which ectopic *ROSEA1* expression is accompanied by strong anthocyanin-pathway activation and broader metabolic and transcriptional changes that may compromise regeneration competence, with altered expression of *PLT5*, *WUS*, and *LBD16* representing one associated feature of this state (Figure 3). Within this framework, metabolic burden, transcription-factor pleiotropy, and possible hormone-related effects should be regarded as interconnected but still untested mechanistic hypotheses.

### 3.2. Broad Functional Similarity of the Regeneration Penalty Across Species

The regeneration penalty associated with *ROSEA1* extended across three eudicot families, namely Solanaceae (tomato and petunia; Figure 1, Figure 2 and Figure 5), Begoniaceae (begonia; Figure 6a–d), and Asteraceae (marigold; Figure 6e–h), indicating a broadly similar phenotype across diverse species rather than a species-specific anomaly. Because the experimental systems differed, however, these comparisons should be interpreted cautiously: tomato and petunia were evaluated primarily in stable transgenic lines, whereas begonia and marigold were assessed in transformation-stage explant assays. In addition, the transcriptional evidence and DELox comparison were obtained only in tomato. This distinction is reinforced by the DELox comparison, which demonstrates that moderate anthocyanin enhancement does not necessarily compromise organogenesis (Figure S1). These results support a regulator- and context-dependent model in which the developmental cost is determined by regulator identity, expression level, and endogenous partner context.

The magnitude of the regeneration penalty appeared to vary across species, ranging from approximately 30% in marigold under moderate cytokinin conditions to nearly 70% in begonia (Figure 6). This variation may reflect differences in baseline regenerative capacity, the endogenous availability of bHLH and WD40 partners that may be needed to assemble functional MBW complexes with *ROSEA1* [12], and species-specific differences in phenylpropanoid metabolism. Genotype-specific responses within marigold further suggest this complexity. In ‘Bonanza Yellow’, which showed relatively low baseline regeneration, no significant penalty was detected (Figure S3), possibly because of a floor effect. In contrast, genotypes with higher regenerative capacity tended to show a clearer *ROSEA1*-associated reduction in regeneration (Figure 6e–h). This pattern is consistent with the well-established genotype dependence of regeneration capacity [23,25] and may also suggest genotype-by-marker-system interactions, with the developmental burden associated with *ROSEA1* potentially becoming more evident in genotypes with moderate to high regenerative potential.

### 3.3. Practical Implications for Transformation Pipeline Design

The distinctive purple pigmentation induced by *ROSEA1* enables the rapid, non-destructive identification of transformed sectors under ambient lighting, offering clear advantages over β-glucuronidase (GUS), which requires destructive histochemical assays, and green fluorescent protein (GFP), which often necessitates specialized fluorescence imaging equipment [2,3,5,52]. Our findings do not diminish these advantages but indicate that they can be offset by reduced regeneration efficiency, particularly in systems with moderate regenerative competence (Figure 1, Figure 2, Figure 5 and Figure 6). Importantly, this cost is not an inherent consequence of anthocyanin production per se. The DELox comparison demonstrates that increased pigment output does not necessarily impair regeneration (Figure S1), indicating that regulator identity and expression context are key determinants of whether a developmental penalty arises.

Several mitigation strategies emerge from these findings. Hormone optimization, particularly increased cytokinin, may partially or, in some contexts, fully offset the apparent phenotype, as demonstrated in marigold (Figure 6e–h and Figure S3), consistent with the broader principle that regeneration barriers can often be alleviated by adjusting the hormonal environment [46]. Co-expression of *PLT5* partially rescued the ROSox regeneration defect while only modestly reducing anthocyanin accumulation (Figure 4), suggesting that reinforcement of developmental competence may provide a useful complementary strategy. Inducible or tissue-specific expression systems, such as the XVE promoter [53], could delay *ROSEA1* activation until after regeneration is established. Alternative reporters, such as *RUBY*, which drives betalain rather than anthocyanin pigmentation, may impose a distinct developmental burden [54]. Finally, genotype pre-screening may also be helpful, because the *ROSEA1*-associated penalty is most pronounced in genotypes with intermediate regeneration capacity; selecting highly regenerable backgrounds can help maintain both visual selection efficiency and acceptable transformation frequencies.

More broadly, these findings highlight that strong constitutive visual-marker systems can intersect with developmental programs during regeneration. The coordinated reduction in *PLT5, WUS,* and *LBD16* expression (Figure 3) may arise from direct transcriptional effects, indirect metabolic perturbation, or a combination of both. Dissecting these mechanisms will be essential for designing next-generation visual reporters that retain strong pigmentation while minimizing developmental cost. Comparative analysis using different anthocyanin regulators under matched expression systems will be particularly informative for distinguishing pigment-associated effects from regulator-specific pleiotropy.

### 3.4. Limitations of the Current Study

Several limitations should be considered when interpreting the mechanistic and cross-system implications of this study. Although the tomato transcriptional data and *PLT5* co-expression experiments provide useful mechanistic clues, the present dataset does not directly test the proposed contributions of metabolic burden, broader transcriptional pleiotropy, or hormone-related effects through global transcriptome-wide profiling (e.g., RNA-seq); hormone quantification; metabolite or metabolic flux analysis; auxin/cytokinin response-marker assays; or *ROSEA1*-related measurements. Because *ROSEA1* is a transcriptional regulator rather than a metabolic endpoint, the present findings should also be generalized cautiously and are best limited to *ROSEA1*-based visual selection rather than to anthocyanin markers as a class. In the stable transgenic systems, insertion sites, transgene copy number, and clonal variation were not directly assessed, so these factors cannot be excluded as contributors to line-to-line differences, particularly in petunia. In the begonia and marigold transformation-stage assays, the observed phenotype is most appropriately interpreted as reduced regenerative output in *ROSEA1*-expressing explants, because the present study does not fully distinguish among reduced explant survival, altered transformation-stage progression, and reduced organogenic competence. In addition, although the discussion draws on relevant published comparisons, some external systems differ from the regeneration-stage assays examined here and should therefore be interpreted as supportive context rather than direct equivalence.

## 4. Materials and Methods

### 4.1. Plant Material and Growth Conditions

Seeds of tomato (*Solanum lycopersicum* cv. ‘Micro-Tom’), petunia (*Petunia hybrida* cv. ‘Mitchell’), and marigold (*Tagetes erecta* cv. ‘Marvel II Yellow’ and *Tagetes patula* cv. ‘Bonanza Yellow’) were purchased from Ball Horticultural Company (West Chicago, IL, USA). *Begonia* ‘UF183-11’, an advanced breeding line developed at the University of Florida, served as plant material. Seeds were surface-sterilized by immersion in 75% (*v*/*v*) ethanol for 1 min, followed by 15% (*v*/*v*) commercial bleach (6% sodium hypochlorite) for 10 min with gentle agitation, and then rinsed six times with sterile distilled water. Sterilized seeds were germinated on Murashige and Skoog (MS) basal medium supplemented with 3% (*w*/*v*) sucrose and solidified with 0.8% (*w*/*v*) agar, pH adjusted to 5.8. Cultures were maintained at 25 °C under a 16 h light/8 h dark photoperiod (100 µmol m^−2^ s^−1^) in a controlled environment chamber. For begonia, mature leaves from greenhouse-grown stock plants were washed under running tap water for 30 min, then surface-sterilized in a laminar flow hood with 70% (*v*/*v*) ethanol for 1 min, followed by 1.5% (*v*/*v*) sodium hypochlorite for 10 min, and rinsed three to five times with sterile distilled water. For marigold, seeds were pretreated for 2 h in a solution containing Contrex AP detergent (4.0 mg L^−1^) and Dithane M-45 fungicide (4 mg L^−1^), rinsed with sterile distilled water, and then surface-sterilized in a laminar flow hood with 20% (*v*/*v*) Clorox (7.5% sodium hypochlorite) plus two drops of Tween 20 for 20 min, followed by 10% (*v*/*v*) Clorox for 10 min. After five rinses with sterile distilled water, seeds were germinated on hormone-free MS medium, and cotyledons from 4-to-5-day-old seedlings were used as explants for regeneration experiments.

### 4.2. Vector Construction

The *ROSEA1* (GenBank: AKB94073.1) and *DELILA* (GenBank: QSC88242.1) coding sequences from *Antirrhinum majus* were synthesized (Gene Universal Inc., Newark, DE, USA) and ligated into the pOX135 binary vector backbone under the control of the CaMV 35S promoter for constitutive expression [55]. For transformation experiments comparing *ROSEA1*-expressing (ROSox), *DELILA*-overexpressing (DELox), and control (CK) treatments, the empty vector lacking the *ROSEA1* or *DELILA* insert served as the control. To generate stable overexpression lines, the same 35S::*ROSEA1* and 35S::*DELILA* constructs were used. All constructs carried *nptII* and a *GFP* reporter cassette driven by the CsVMV promoter, with *nptII* conferring kanamycin resistance. Construct integrity was verified by Sanger sequencing before transformation into *Agrobacterium tumefaciens* strain EHA105. The coding region of *PLT5* (AT5G57390) was synthesized (Gene Universal Inc., Newark, DE, USA). For co-expressing *PLT5* and *ROSEA1* (P + ROSox), full 2 × CaMV35S::DR::HSter cassettes were amplified from the corresponding single-expressing vectors and sequentially assembled into pOX135 using the AatII/MluI sites, following our previous strategy [55,56].

### 4.3. Agrobacterium-Mediated Transformation

*Agrobacterium tumefaciens* strain EHA105 harboring the binary vectors was cultured in 25 mL LB broth supplemented with spectinomycin (100 mg L^−1^) and rifampicin (50 mg L^−1^) at 28 °C with shaking at 180 rpm overnight. A secondary culture was initiated by transferring 1 mL of the overnight culture into 10 mL of fresh LB broth containing the same antibiotics and growing it until the optical density at 600 nm (OD_600_) reached 0.3–0.5, except for marigold, for which cultures were grown to OD_600_ = 0.6–1.0. Bacterial cells were pelleted by centrifugation and resuspended in liquid MS medium supplemented with 100 µM acetosyringone for plant infection [56].

For tomato transformation, cotyledon explants excised from 7-to-10-day-old seedlings were immersed in the *Agrobacterium* suspension for 10 min with gentle agitation. Inoculated explants were blotted on sterile filter paper and co-cultivated on MS medium (MS salts and vitamins, 30 g L^−1^ sucrose, 2.5 g L^−1^ Phytagel, pH 5.8) in the dark at 25 °C for 2 days. Explants were then transferred to callus induction medium (CIM) consisting of MS medium supplemented with 2 mg L^−1^ zeatin, 0.2 mg L^−1^ NAA, 100 mg L^−1^ kanamycin, and 100 mg L^−1^ timentin. Cultures were maintained for 2–5 weeks with subculturing onto fresh CIM every 2 weeks. Regenerating shoots were subsequently transferred to shoot induction medium (SIM) composed of MS medium with 1 mg L^−1^ zeatin, 100 mg L^−1^ kanamycin, and 100 mg L^−1^ timentin, followed by rooting on hormone-free MS medium containing 100 mg L^−1^ kanamycin and 100 mg L^−1^ timentin. Regenerated shoots exhibiting purple pigmentation, indicative of anthocyanin accumulation, were selected and transferred to a rooting medium. Ten rooted plantlets were acclimatized in a growth chamber and transferred to soil. For tomato, three independent T_0_ transgenic plants for each construct were confirmed by PCR amplification of the GFP transgene. Seeds from confirmed T_0_ plants were germinated on kanamycin-containing medium to select T_1_ progeny, and one representative independent T_1_ line from each construct was chosen for subsequent regeneration assays. In petunia, the same procedure was followed, except that the two representative independent T_1_ lines were selected for subsequent assays [57].

For petunia transformation, cotyledon explants from 7-to-10-day-old seedlings were inoculated as described above and co-cultivated on MS medium in the dark at 25 °C for 2 days. Explants were then transferred to CIM comprising MS medium supplemented with 1 mg L^−1^ BAP, 0.1 mg L^−1^ NAA, 100 mg L^−1^ kanamycin, and 100 mg L^−1^ timentin. Because shoot regeneration occurred directly on CIM without a distinct induction phase, explants were maintained on the same medium for 2–4 weeks with subculturing every 2 weeks until regenerated shoots were sufficiently developed for transfer to hormone-free MS medium containing 100 mg L^−1^ kanamycin and 100 mg L^−1^ timentin for elongation and rooting [58].

For begonia transformation, leaf segments (approximately 0.5 × 0.5 cm) from sterile in vitro culture were inoculated with the *Agrobacterium* suspension and co-cultivated on regeneration medium (REM), consisting of MS medium supplemented with 1.5 mg L^−1^ TDZ and 0.375 mg L^−1^ NAA, in the dark at 25 °C for 2 days. The explants were then transferred to REM supplemented with 75 mg L^−1^ kanamycin and 100 mg L^−1^ timentin. Cultures were subcultured every 2 weeks. After 4–9 weeks, regenerating explants were transferred to hormone-free MS medium with 75 mg L^−1^ kanamycin and 100 mg L^−1^ timentin to promote shoot elongation and rooting [55].

For marigold transformation, cotyledon explants from 4-to-5-day-old seedlings were vacuum-infiltrated with the *Agrobacterium* suspension for 5–10 min to enhance T-DNA delivery, then co-cultivated on MS medium in the dark at 25 °C for 2 days. To evaluate the effect of cytokinin concentration on regeneration under *ROSEA1* expression, three CIM formulations differing only in BAP level were tested, namely CIM1 (MS medium + 0.5 mg L^−1^ NAA + 0.75 mg L^−1^ BAP), CIM2 (MS medium + 0.5 mg L^−1^ NAA + 2.0 mg L^−1^ BAP), and CIM3 (MS medium + 0.5 mg L^−1^ NAA + 2.5 mg L^−1^ BAP), all supplemented with 100 mg L^−1^ kanamycin and 100 mg L^−1^ timentin. After callus formation, explants from each CIM treatment were transferred to a corresponding shoot induction medium (SIM: MS + NAA 0.1 mg L^−1^) at the respective BAP concentration to maintain hormonal continuity during regeneration. Regenerated shoots were subsequently transferred to hormone-free MS medium containing 200 mg L^−1^ kanamycin and 100 mg L^−1^ timentin for elongation and rooting. Unless stated otherwise, all basal media consisted of MS salts and vitamins, 30 g L^−1^ sucrose, and 7 g L^−1^ agar (pH 5.8).

### 4.4. Regeneration Assays

Two categories of regeneration assay were conducted throughout this study: transformation-stage assays, in which freshly inoculated explants were tracked during *Agrobacterium*-mediated transformation, and stable-line assays, in which explants derived from confirmed transgenic lines (e.g., ROSox, DELox, or P + ROSox, depending on the experiment) were compared with the corresponding control or reference lines under identical culture conditions. Because all quantitative comparisons of regeneration capacity reported in this work originate from these assays, the scoring criteria and experimental design are described in detail below.

In transformation-stage assays, explants were cultured on the species-specific CIM formulations described above. Regeneration was scored at discrete time points rather than across continuous intervals: at weeks 2, 3, and 4 for tomato; at weeks 1, 2, and 3 for petunia; at weeks 3, 5, 7, and 9 for begonia; and at weeks 2, 4, 6, and 8 for marigold. In stable-line assays, leaf or cotyledon explants excised from greenhouse-grown ROSox and CK plants were placed on the corresponding regeneration medium and cultured under the same light, temperature, and subculture regime used for transformation-stage experiments.

Regeneration capacity was evaluated using two complementary metrics. Regeneration frequency was defined as the percentage of explants that produced at least one visible shoot ≥ 5 mm in length by a given scoring date. Shoots per explant were calculated as the total number of shoots (≥5 mm) divided by the total number of explants in that treatment. In addition, explant area was measured from calibrated overhead photographs at each time point using ImageJ v1.54u7 (NIH, Bethesda, MD, USA) to provide a quantitative record of tissue expansion independent of shoot formation.

A biological replicate was defined as one independently prepared culture plate (Petri dish) containing 15–20 explants from the same treatment/genotype. Each treatment included a minimum of 15 explants per biological replicate, and at least three independent biological replicates were performed for every experiment unless otherwise noted. The exact number of explants (n) and replicates for each dataset is reported in the corresponding figure legends.

### 4.5. Anthocyanin Extraction and Quantification

Anthocyanins were extracted following the acidified methanol protocol with modifications. Fresh tissue (50–100 mg) from regenerating explants or callus was weighed, flash-frozen in liquid nitrogen, and homogenized to a fine powder. Samples were extracted in 300 µL of acidified methanol [1% (*v*/*v*) HCl in methanol] by vortexing and incubating overnight at room temperature in the dark. Following extraction, 200 µL of Milli-Q water and 500 µL of chloroform were added to facilitate phase separation. Samples were vortexed vigorously and centrifuged (12,000× *g*, 5 min, 4 °C). A 350 µL aliquot of the upper aqueous-–methanolic phase was transferred to a fresh tube and diluted to a final volume of 1.05 mL with 415.8 µL methanol, 4.2 µL concentrated HCl, and 280 µL Milli-Q water. Absorbance was measured at 530 nm (anthocyanin peak) and 657 nm (chlorophyll interference) using a UV-visible spectrophotometer (NanoDrop 2000; Thermo Fisher Scientific, Wilmington, DE, USA). Corrected anthocyanin absorbance was calculated as Acorr = A_530_ − 0.25 × A_657_. Anthocyanin content was expressed as milligrams of cyanidin-3-glucoside equivalents per gram fresh weight (mg g^−1^ FW) using the molar extinction coefficient ε = 33,000 L mol^−1^ cm^−1^ and molecular weight 449.2 g mol^−1^. Blank extractions processed without tissue were included to correct for background absorbance.

### 4.6. RNA Isolation and Quantitative Real-Time PCR

For the qRT-PCR analyses reported in this study, regenerating tissues were collected from stable transgenic tomato ROSox lines and corresponding CK lines at weeks 2 and 3 after regeneration induction. Total RNA was extracted from 80 to 100 mg of flash-frozen regenerating tissue using RNAzol RT reagent (Molecular Research Center, Cincinnati, OH, USA) following the manufacturer’s protocol. RNA concentration and purity were assessed using a NanoDrop 2000 spectrophotometer; samples with A_260_/A_280_ ratios between 1.9 and 2.1 were used for cDNA synthesis. Genomic DNA contamination was eliminated by on-column DNase I digestion using the RNase-Free DNase Set (QIAGEN, Hilden, Germany). First-strand cDNA was synthesized from 1 µg of DNase-treated RNA using the QuantiTect Reverse Transcription Kit (QIAGEN, Hilden, Germany). The resulting cDNA was diluted 20- to 25-fold for use as qPCR template.

Quantitative real-time PCR (qRT-PCR) was performed on a CFX96 Touch Real-Time PCR Detection System (Bio-Rad, Hercules, CA, USA) using iTaq Universal SYBR Green Supermix (Bio-Rad, Hercules, CA, USA). Each 10 µL reaction contained 5 µL of 2× SYBR Green master mix, 4 µL of diluted cDNA template, and 0.5 µL of a 10 µM forward and reverse primer mixture. Gene-specific primers were designed using web-based Primer-BLAST (NCBI, Bethesda, MD, USA) to span exon–exon junctions where possible and to generate amplicons of 100–150 bp with melting temperatures of 58–62 °C. Target genes included *ROSEA1* (transgene expression), anthocyanin biosynthetic genes (*CHI*, chalcone isomerase; *F3H*, flavanone 3-hydroxylase; *F3′5′H*, flavonoid 3′,5′-hydroxylase; *DFR*, dihydroflavonol 4-reductase), and regeneration-associated transcription factors (*PLT5*, *PLETHORA5*; *WUS*, *WUSCHEL*; *LBD16*, *LATERAL ORGAN BOUNDARIES DOMAIN 16*). These regeneration-associated regulators were selected based on their established roles in pluripotency acquisition and shoot meristem identity during de novo organogenesis. Primer sequences are provided in Supplementary Table S1. Thermal cycling conditions consisted of initial denaturation at 95 °C for 3 min, followed by 40 cycles of 95 °C for 10 s and 60 °C for 30 s. Melt curve analysis was performed to verify primer specificity. Relative gene expression was calculated using the 2^−ΔΔCt^ method with *ACTIN2* as the internal reference gene. Three biological replicates, each comprising three technical replicates, were analyzed per genotype and time point.

### 4.7. Imaging and Microscopy

Macroscopic images of regenerating explants and culture plates were captured using a digital camera under standardized lighting conditions. To confirm transgene expression and T-DNA integration, explants were visualized using a fluorescence stereomicroscope equipped with appropriate filter sets. For GFP observation in begonia and marigold, signals were detected using a Leica DM6000 B fluorescent microscope (Leica Microsystems GmbH, Wetzlar, Germany) equipped with a GFP filter set, following the general procedure described in Jiang et al. [55]. Purple pigmentation indicative of anthocyanin accumulation was recorded at each time point. In addition, anthocyanin extracts were photographed to illustrate differences in color intensity between genotypes.

### 4.8. Statistical Analysis

All data visualization and statistical analyses were performed using GraphPad Prism 10 (version 10.4.2). Regeneration experiments were conducted with *n* = 4 biological replicates, each consisting of 20 explants per treatment/genotype, unless otherwise stated. Biochemical and gene expression analyses were performed using three independent biological replicates. For qRT-PCR, each biological replicate was analyzed with three technical replicates. Morphological traits were quantified from a minimum of ten biological replicates per genotype. Data are presented as mean ± s.d. Statistical significance was assessed using two-tailed Student’s *t*-tests for pairwise comparisons. For comparisons involving more than two groups, including the analyses in Figure 5, one-way ANOVA was performed at each time point, followed by Tukey’s multiple-comparisons test. *p* < 0.05 was considered statistically significant. Exact *p*-values and sample sizes are indicated in the corresponding figure legends.

## 5. Conclusions

Constitutive *ROSEA1* expression was associated with reduced regeneration across the plant systems examined in this study, including both stable transgenic lines and transformation-stage assays. In tomato, this phenotype was accompanied by increased anthocyanin accumulation and reduced expression of key regeneration-associated regulators during early regeneration, while *PLT5* co-expression partially alleviated the phenotype. Taken together, these observations identify a reproducible *ROSEA1*-associated regeneration penalty under constitutive expression, while indicating that the current mechanistic evidence remains correlative. The severity of this phenotype varied with experimental context, including species, genotype, and hormone regime.

## Data Availability

All data supporting the findings of this study are available from the corresponding author upon reasonable request.

## Supplementary Materials

The following supporting information can be downloaded at https://www.mdpi.com/article/10.3390/plants15132004/s1. Figure S1: Stable transgenic tomato lines overexpressing *DELILA* do not show an obvious regeneration penalty under the conditions tested. a, Representative regeneration phenotypes of control (CK) and *DELILA* overexpression (DELox) tomato lines during *in vitro* culture. Scale bars, 1 cm. b, Anthocyanin content (mg g^−1^ FW) in CK and DELox tissues at week 3 (*n* = 4 biological replicates; three technical replicates per biological replicate). c, Shoots per explant in CK and DELox lines at week 3. Data are presented as mean ± s.d. (*n* = 5 biological replicates; 20 explants per replicate). Statistical significance was assessed using two-tailed Student’s *t*-tests; *p*-values are indicated. Figure S2: Repeated trials show a consistent reduction in regenerative output in stable transgenic petunia lines overexpressing *ROSEA1*. a, Time-course of regeneration from week 1 to week 3 in petunia explants for control (CK, top) and *ROSEA1* overexpression (ROSox-2 in Figure 5, bottom) lines, showing reduced plantlet formation in ROSox. b, Representative close-up views of regenerating tissues at week 2 and week 3 for CK and ROSox. c, Explant area quantified from week 1 to week 3 (CK, green; ROSox-2, purple) (*n* = 20 explants measured per genotype at each time point). d, Regeneration frequency (%) at week 2 and week 3 in CK and ROSox-2 lines (*n* = 4 biological replicates; 20 explants per replicate). e, Representative anthocyanin extracts from CK and ROSox tissues collected at week 3. f, Anthocyanin content (mg g^−1^ FW) at week 3 in CK and ROSox tissues (*n* = 4 biological replicates; 3 technical replicates per biological replicate). Data are presented as mean ± s.d. Statistical significance was assessed using two-tailed Student’s *t*-tests; *p*-values are indicated. Scale bars, 1 cm (a) and 5 mm (b). Figure S3: The *ROSEA1*-associated regeneration phenotype is not detectable in marigold under high-cytokinin conditions or in the low-regenerating genotype ‘Bonanza Yellow’. a, Representative Petri dishes of ‘Marvel II Yellow’ explants under control (CK, left) and *ROSEA1* (ROSox, right) treatments. b,c, Regeneration frequency (%) (b) and shoots per explant (c) for ‘Marvel II Yellow’ (CK, green; ROSox, purple). d, Representative Petri dishes of ‘Bonanza Yellow’ explants under CK (left) and ROSox (right) treatments on CIM1. e,f, Regeneration frequency (%) (e) and shoots per explant (f) for ‘Bonanza Yellow’ (CK, green; ROSox, purple). Data are presented as mean ± s.d. (*n* = 4 biological replicates; 15 explants per replicate). Statistical significance was assessed using two-tailed Student’s *t*-tests; *p*-values are indicated. Scale bars, 1 cm (a,d). Table S1: List of primers used for qRT-PCR analysis.

## Abbreviations

The following abbreviations are used in this manuscript:

*PLT5*	PLETHORA 5
*WUS*	WUSCHEL
*LBD16*	LATERAL ORGAN BOUNDARIES DOMAIN 16
*CHI*	chalcone isomerase
*F3H*	flavanone 3-hydroxylase
*F3′5′H*	flavonoid 3′,5′-hydroxylase
*DFR*	dihydroflavonol 4-reductase
