# Supplementary material for: ROSEA1-Based Visual Selection Reduces Plant Regeneration and Alters Developmental Regulator Expression
Source: Plants (Basel). 2026 Jun 28;15(13):2004. doi: 10.3390/plants15132004 (PMC13364241; doi:10.3390/plants15132004)
Supplement: Supplementary file 1 [file plants-15-02004-s001.zip › plants-4373424-supplementary.pdf]

Supplementary materials:

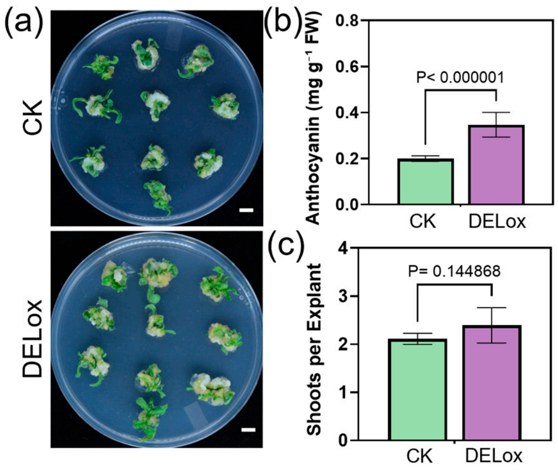

Supplementary Figure S1 Stable transgenic tomato lines overexpressing *DELILA* do not show an obvious regeneration penalty under the conditions tested. a, Representative regeneration phenotypes of control (CK) and *DELILA* overexpression (DELox) tomato lines during *in vitro* culture. Scale bars, 1 cm. b, Anthocyanin content (mg g^{-1} FW) in CK and DELox tissues at week 3 (n = 4 biological replicates; three technical replicates per biological replicate). c, Shoots per explant in CK and DELox lines at week 3. Data are presented as mean ± s.d. (n = 5 biological replicates; 20 explants per replicate). Statistical significance was assessed using two-tailed Student's *t*-tests; *P*-values are indicated.

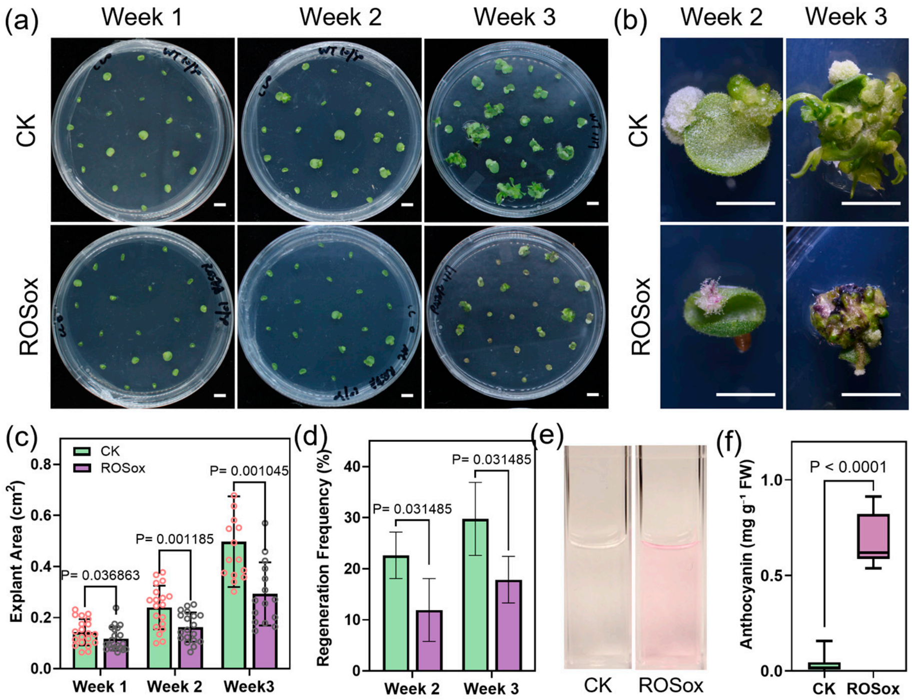

Supplementary Figure S2 Repeated trials show a consistent reduction in regenerative output in stable transgenic petunia lines overexpressing *ROSEA1*. a, Time-course of regeneration from week 1 to week 3 in petunia explants for control (CK, top) and *ROSEA1* overexpression (ROSox-2 in Fig. 5, bottom) lines, showing reduced plantlet formation in ROSox. b, Representative close-up views of regenerating tissues at week 2 and week 3 for CK and ROSox. c, Explant area quantified from week 1 to week 3 (CK, green; ROSox-2, purple) (n = 20 explants measured per genotype at each time point). d, Regeneration frequency (%) at week 2 and week 3 in CK and ROSox-2 lines (n = 4 biological replicates; 20 explants per replicate). e, Representative anthocyanin extracts from CK and

ROSox tissues collected at week 3. f, Anthocyanin content (mg g^{-1} FW) at week 3 in CK and ROSox tissues (n = 4 biological replicates; 3 technical replicates per biological replicate). Data are presented as mean ± s.d. Statistical significance was assessed using two-tailed Student's *t*-tests; *P*-values are indicated. Scale bars, 1 cm (a) and 5 mm (b).

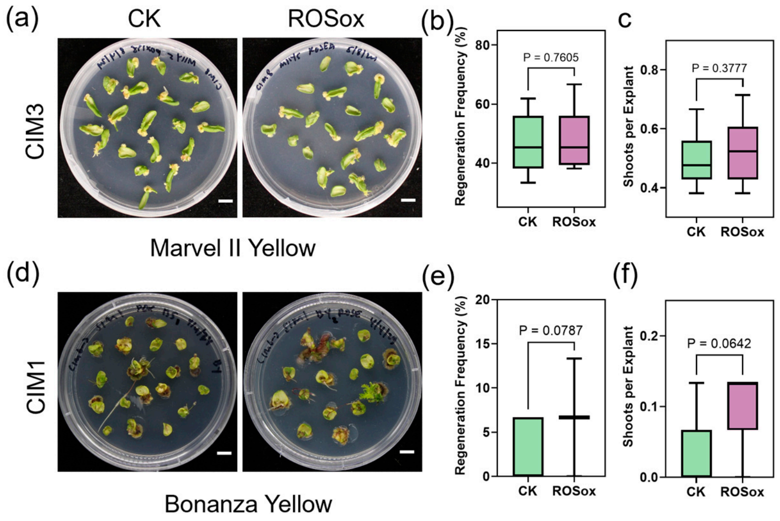

Supplementary Figure S3 The *ROSEA1*-associated regeneration phenotype is not detectable in marigold under high-cytokinin conditions or in the low-regenerating genotype 'Bonanza Yellow'. a, Representative Petri dishes of 'Marvel II Yellow' explants under control (CK, left) and *ROSEA1* (ROSox, right) treatments. b,c, Regeneration frequency (%) (b) and shoots per explant (c) for 'Marvel II Yellow' (CK, green; ROSox, purple). d, Representative Petri dishes of 'Bonanza Yellow' explants under CK (left) and ROSox (right) treatments on CIM1. e,f, Regeneration frequency (%) (e) and shoots per explant (f) for 'Bonanza Yellow' (CK, green; ROSox, purple). Data are presented as mean ± s.d. (n = 4 biological replicates; 15 explants per replicate). Statistical significance was assessed using two-tailed Student's *t*-tests; *P*-values are indicated. Scale bars, 1 cm (a,d).

Supplementary Table S1 List of primers used for qRT-PCR analysis

Gene name	Forward primer sequence （5'→3'）	Reverse primer sequence（5'→3'）	Note
SlACT2	TTGCTGACCGTATGAGCAAG	GGACAATGGATGGACCAGAC	qRT-PCR in tomato
ROSEA1	ACGTTACTTGGCCGAGAGAA	AACTTCATCTTGTGGCGACG	
SlCHI	TGCAACAATGGAAGGGCAAA	CGACTTCTGCTAAGCGATCG	
SlF3H	CGGTTTGACATGTCTGGTGG	CGCCAGTCTTGAACCACTTC	
SlF3′5′H	GGTTCAATGCCACATGTTGC	GCAACAACCATCCCACATGT	
SlDFR	GCTGGAGCGATTTGGACTTC	CAGCCTTCTCTGCCAGTATCTT	
SlPLT5	GTGGCTTGGCTTCTCACTTT	GCTTGGAAGAGGGAAAGTTGT	
SlWUS	TGTGCAGGCAAAGTAGTAGC	TGTTCAGCAGTTGGAGACCT	
SlLBD16	TGTTACACGTTCCTGTCCCC	AACTTTGTGCCAGCTGAGTC	
GFP	ACAAGTTCAGCGTGTCCG	TCACCTTGATGCCGTTCT	PCR
